# Does Sb_2_Se_3_ Admit Nonstoichiometric Conditions? How Modifying the Overall Se Content Affects the Structural, Optical, and Optoelectronic Properties of Sb_2_Se_3_ Thin Films

**DOI:** 10.1021/acsami.1c20764

**Published:** 2022-03-01

**Authors:** Ivan Caño, Pedro Vidal-Fuentes, Lorenzo Calvo-Barrio, Xavier Alcobé, José Miguel Asensi, Sergio Giraldo, Yudania Sánchez, Zacharie Jehl, Marcel Placidi, Joaquim Puigdollers, Victor Izquierdo-Roca, Edgardo Saucedo

**Affiliations:** † Escola d’Enginyeria de Barcelona Est (EEBE), Universitat Politècnica de Catalunya, Av. Eduard Maristany, 16, 08019 Barcelona, Spain; ‡ Institut de Recerca en Energia de Catalunya (IREC), Jardins de les Dones de Negre, 1, 08930 Sant Adrià del Besòs, Spain; § Centres Científics i Tecnològics (CCiTUB), Universitat de Barcelona, C. Lluis Solé i Sabaris 1-3, 08028 Barcelona, Spain; ∥ IN2UB, Departament d′Enginyeria Electrònica i Biomèdica, Universitat de Barcelona, C. Martí i Franquès, 1, 08028 Barcelona, Spain; ⊥ Departament de Física Aplicada, Universitat de Barcelona, C. Martí i Franquès, 1, 08028 Barcelona, Spain

**Keywords:** quasi-1D semiconductors, chalcogenides, photovoltaics, Sb_2_Se_3_, MoSe_2_, material characterization, emerging materials

## Abstract

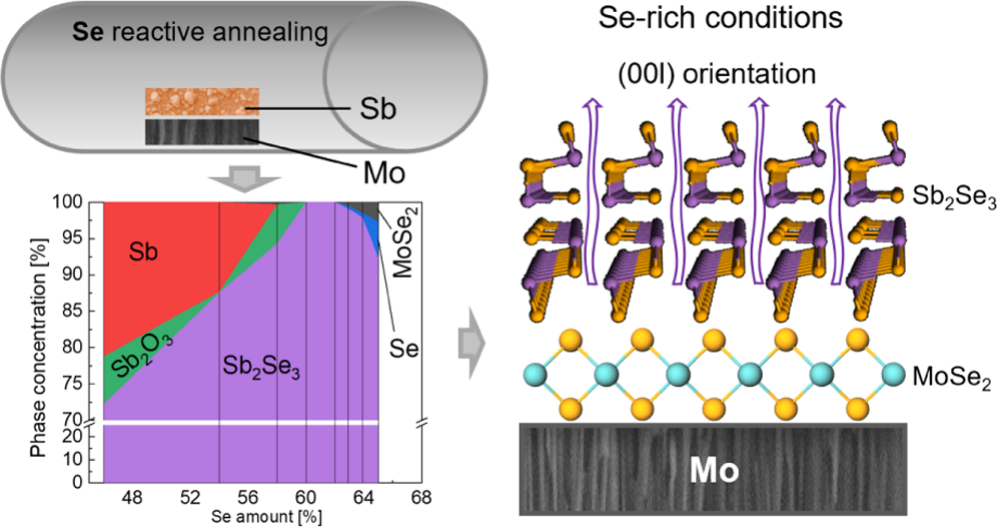

Sb_2_Se_3_ is a quasi-one-dimensional (1D) semiconductor, which has shown great promise in photovoltaics. However, its performance is currently limited by a high *V*_oc_ deficit. Therefore, it is necessary to explore new strategies to minimize the formation of intrinsic defects and thus unlock the absorber’s whole potential. It has been reported that tuning the Se/Sb relative content could enable a selective control of the defects. Furthermore, recent experimental evidence has shown that moderate Se excess enhances the photovoltaic performance; however, it is not yet clear whether this excess has been incorporated into the structure. In this work, a series of Sb_2_Se_3_ thin films have been prepared imposing different nominal compositions (from Sb-rich to Se-rich) and then have been thoroughly characterized using compositional, structural, and optical analysis techniques. Hence, it is shown that Sb_2_Se_3_ does not allow an extended range of nonstoichiometric conditions. Instead, any Sb or Se excesses are compensated in the form of secondary phases. Also, a correlation has been found between operating under Se-rich conditions and an improvement in the crystalline orientation, which is likely related to the formation of a MoSe_2_ phase in the back interface. Finally, this study shows new utilities of Raman, X-ray diffraction, and photothermal deflection spectroscopy combination techniques to examine the structural properties of Sb_2_Se_3_, especially how well-oriented the material is.

## Introduction

In recent years, thin-film chalcogenide-based photovoltaic (PV) technologies have emerged as one of the most attractive complementary pathways to silicon-based solar cells, opening the door to greater versatility and implementation opportunities for photovoltaics, including flexible and semitransparent devices. Among these innovative absorbers, CdTe and Cu(In,Ga)(S,Se)_2_ (CIGS) have accomplished remarkable improvements in their power conversion efficiency (PCE), exceeding the 20% barrier.^1,2^ Nevertheless, the potentially unstable supply of scarce elements such as In, Ga, and Te and the toxicity of Cd raise doubts about the ability of CIGS and CdTe to meet the necessary industrial demands to transition toward a 100% renewable energy system.^3−5^ Consequently, thin-film chalcogenide-based photovoltaics free of critical raw materials remain a highly active research field, generating relevant scientific and technological awareness. For instance, Cu_2_SnZn(S,Se)_4_ (CZTSSe) has aroused great interest as a successful sustainable alternative to CIGS, achieving PCE values of up to 12.6%.^6^ However, its chemical complexity makes it difficult to overcome the current limitations, mostly regarding the high number of native defects and secondary phases.^7−9^

In contrast, antimony selenide (Sb_2_Se_3_) is constituted by earth-abundant and low-toxicity components and possesses a number of highly appealing properties for photovoltaic implementation, such as binary stoichiometry, an optimal band gap (1.2–1.3 eV), a large absorption coefficient, and a quasi-one-dimensional (Q-1D) crystal structure.^10^ In addition, solar cells made with this compound can be fabricated in both substrate and superstrate configurations^11,12^ at comparatively low temperatures,^13^ thus paving the way for alternative substrates and architectures. Furthermore, Sb_2_Se_3_ has shown great promise not only for photovoltaics but also for energy storage, achieving a high reversible capacity of 312.03 mAh/g at a current density of 1000 mA/g for potassium-ion batteries (PIBs).^14,15^ The Q-1D structure of Sb_2_Se_3_ consists of (Sb_4_Se_6_)*_n_* covalently bonded ribbons along one crystallographic direction, while they are stacked by weak van der Waals forces in the other two directions,^16^ conferring strong anisotropic optoelectronic properties, such as preferential carrier transport in the [001] direction (along the covalently bonded ribbons according to *Pbnm* #62). Hence, by tuning the crystalline orientation of the Sb_2_Se_3_ film, it is possible to increase the minority carrier mobility and collection and thus improve the photovoltaic (PV) performance of the device.^17^ Thus far, it has been reported that the substrate on which the Sb_2_Se_3_ is grown has a significant influence on the ribbon alignment, along with the temperature at which the material is subjected during synthesis (with a reported optimal *T* range of 300–330 °C).^18−20^ However, the effect of the relative amount of Se/Sb on the film’s crystalline orientation is yet to be thoroughly investigated.

In a very short time, Sb_2_Se_3_ has shown steady improvements in the PV performance, achieving record cell efficiencies of 9.2% (nanorod arrays) and 8.5% (planar heterojunction solar cells), prepared by close-spaced sublimation,^11,21^ and 6.84% by magnetron sputtering.^22,23^ However, devices are still mainly limited by a high *V*_oc_ deficit, suggesting that recombination processes involving deep defects might be a limiting factor.^24,25^ Indeed, despite being a relatively simple binary compound, it has been shown by first-principles calculations that the intrinsic defects in Sb_2_Se_3_ are unexpectedly complicated and unconventional, with up to three different *V*_Se_ and two *V*_Sb_ components due to several nonequivalent atomic sites, in addition to substitutional and intrinsic defects and other uncommon defects, such as 2Se replacing one Sb antisite (2Se_Sb_).^26^ There is consequently a clear interest in exploring novel strategies that make it possible to control the emergence of these defects and to offer new conditions to unlock the whole potential of the absorber.

In particular, modulating the relative amount of Se/Sb by moving away from the stoichiometric conditions could offer an opportunity to compensate defects, while modifying the electronic properties of the material, without adding new components (i.e., complexity) to the system. For instance, according to Huang et al.,^26^ the electrical conductivity could be tuned from p-type to n-type by modulating the Se chemical potential through transitioning from Se-rich to Se-poor conditions. Moreover, it has been reported by theoretical studies that deep defects have a higher concentration under Sb-rich conditions, suggesting that a hypothetical strategy to enhance the performance of the device could be by favoring a Se-rich synthesis.^24^ However, it is still unclear whether Sb_2_Se_3_ allows an extended off-stoichiometric range or, on the contrary, grows forming a single phase highly resilient to compositional variations. Indeed, off-stoichiometric film composition has been a key element in the past success of mature thin-film chalcogenide absorbers such as CIGS or CZTSSe, especially in terms of control of the minority carrier concentration.^27−29^ For example, it has been shown that the shallow defect *V*_Cu_ in kesterites becomes dominant under Cu-poor conditions, contributing to an enhanced p-type conductivity.^30,31^

Considering these relevant characteristics of chalcogenide absorbers such as CIGS and CZTSSe, it is extremely important to investigate the tolerance of Sb_2_Se_3_ to intrinsic doping and compositional variations, moving toward a broader understanding of this material with the goal of developing improvement strategies based on verifiable empirical knowledge.

Remarkably, recent experimental evidence has revealed that moderate Se excess increases the PV performance of Sb_2_Se_3_/CdS p-n junction-based devices.^32,33^ Nonetheless, whether this excess has been incorporated into the structure or segregated in the form of additional phases is still unclear.

Here, we have used a sequential process based on the selenylation of thermally evaporated Sb to synthesize a series of Sb_2_Se_3_ thin films with different Se/Sb relative contents, ranging in a wide spectrum of nominal compositions. By forcing the incorporation of different Se amounts, we expected to elucidate whether the Se and Sb excesses had been incorporated into the Sb_2_Se_3_ structure and thus determine whether Sb_2_Se_3_ is a highly stoichiometric single-phase semiconductor in the same manner as in CdTe or it admits an extended compositional range as it occurs in CIGS or CZTS. Also, we have identified all the phases that appear under the different conditions studied, with which we have been able to develop a phase diagram as a function of the overall Se content. Interestingly, we have detected a correlation between the amount of the self-generated MoSe_2_ phase and the preferential crystalline orientation of Sb_2_Se_3_, proving that the presence of such a phase is critical to obtain good-quality devices with an optimal orientation. Finally, prototype solar cells have been fabricated and characterized to assess the effect of varying the Se content in the absorber on the PV performance of the device.

## Experimental Methodology

### Material and Device Preparation

Sb_2_Se_3_ layers were manufactured on Mo-sputtered SLG substrates (SLG/Mo), using a two-step sequential process consisting of the deposition of a 250 nm Sb layer followed by reactive annealing under a Se atmosphere. Sb coating was performed by thermal evaporation (Oerlikon Univex 250), from Sb shots (Alfa Aesar, 1–3 mm), using a base vacuum of 10^–5^ mbar and an evaporation rate of 1 Å/s. Then, the SLG/Mo/Sb precursors were subjected to Se reactive annealing in a tubular furnace, using a semiclosed graphite box (23 cm^3^). Accordingly, samples were heated up to 320 °C (with a 20 °C/min heating ramp) at 500 mbar and then cooled naturally (45 min approximately). To explore different compositions, different annealing durations were applied between 15 min (Se-poor) and 30 min (Se-rich). Likewise, the Se availability was modulated by changing the amount of Se powder placed in the graphite boxes, without any condition below the threshold required to achieve a saturated atmosphere. To synthesize Sb-rich films (Se contents below its stoichiometric amount), 10–15 mg of Se powders was used (Alfa Aesar, Se powder 200 mesh). On the other hand, to synthesize Se-rich samples, approximately 25 mg was placed in the boxes. The appropriate contents in each case were determined through previous optimization processes. Importantly, graphite boxes are subjected to high-temperature cleaning (650 °C, 2 h) beforehand to eliminate any possible selenium excess in between each sample processing.

To characterize the optoelectronic properties, some of the Sb_2_Se_3_ layers prepared in accordance with the previous procedure were converted into solar cells. The heterojunction was completed with an n-type CdS buffer deposited by chemical bath deposition, followed by i-ZnO + indium tin oxide (ITO) deposition by direct current (DC) pulsed magnetron sputtering (Alliance Concept CT100), as reported elsewhere.^34^

### Material and Device Characterization

The nominal composition and thickness of the as-synthesized Sb_2_Se_3_ absorbers were determined by X-ray fluorescence (XRF) with Fischerscope XVD equipment, which was previously calibrated by inductively coupled plasma mass spectrometry (ICP-MS). Cross-sectional morphology and thickness of complete devices were characterized by scanning electron microscopy (SEM), using a Zeiss Series Auriga field-emission microscope, with an acceleration voltage of 5 kV and working distances ranging between 3 and 5 mm. Composition profiles were studied by X-ray photoelectron spectroscopy (XPS) in the CCiTUB with a PHI 5500 multitechnique system from Physical Electronics, using a monochromatic X-ray source Al Kα line of 1486.6 eV. X-ray diffraction (XRD) data were obtained using a PANalytical X’Pert PRO MPD alpha1 Bragg-Brentano powder diffractometer, with a Cu tube operating at 45 kV and 40 mA, a Johansson-type Ge (111) primary focalizing monochromator, and a solid-state strip 1D PIXcel^1D^ detector. High-resolution, high-statistics, full-angular range Cu Kα_1_ θ/2θ scans were performed with the following parameters: 2θ/θ scans from 4 to 145°; step size of 0.0131°; measuring time per step of 200 s (PIXcel^1D^ active length of 3.347°); three consecutive repeated scans; total measuring time per sample of 7.2 h. An automatic divergence slit system and a mask enabled a constantly irradiated surface (10 × 12 mm^2^) over the analyzed samples. The diffracting volume is also constant regarding the small and finite thickness (below 2 micrometers) of the characterized layers. Full profile analysis has been performed, applying Rietveld refinement for all the crystalline structure phases observed.^35^ The refinements have been performed with TOPAS v6 software.^36^ The peak width of each phase was modeled with the double-Voigt approach by considering both the Lorentzian contribution of the crystallite size effect and the Gaussian contribution of the microstrain to the peak width.^37^ Preferential orientation corrections were applied by spherical harmonics or alternatively by the March–Dollase function.^38^ The background was modeled with a 15th-order Chebyshev polynomial. The instrumental contribution to the diffraction profile was calculated with the fundamental parameters approach.^39^ Raman spectroscopy measurements were performed with an optical probe in the backscattering configuration developed at IREC facilities coupled to a FHR640 Horiba Jobin Yvon spectrometer, where the signal is acquired with a liquid nitrogen-cooled (140 K) CCD detector. The excitation wavelength employed was 633 nm and was focused on a macrospot (∼50 μm) to avoid sample inhomogeneity. The laser power was kept under 25 mW/cm^2^ to avoid degradation of the films.

Additionally, the optical absorption of glass (Corning1737)/Sb_2_Se_3_ samples was characterized by photothermal deflection spectroscopy (PDS). This technique was used to determine the absorptance of the Sb_2_Se_3_ films in the sub-band gap region. A single-slab model was applied to compute the absorption coefficient (see the Supporting Information for a more detailed description of the procedure). The transverse PDS setup used in this work consists of a 100 W tungsten halogen lamp, PTI 01-0002 monochromator (two-grating monochromator, spectral range of 400–2000 nm), and Thorlabs MC1000 optical chopper (4 Hz light modulation frequency). A Signal Recovery 7265 lock-in amplifier was connected to a Hamamatsu C10442-02 PSD position-sensitive detector to measure the deflection of a MC6320C 10 mW laser probe beam. Samples were put in a quartz cell filled with Fluorinert TM FC-40. A personal computer was used to control the monochromator, change the order filters, and store the PDS signal read from the lock-in amplifier.^40^

SLG/Mo/Sb_2_Se_3_/CdS/ZnO/ITO devices were characterized by current density–voltage measurements (*J*–*V* curves), using a Sun 3000 AAA-class Abet solar simulator, with a uniform illumination area of 15 × 15 cm^2^, calibrated with a Si reference solar cell. Optoelectronic characterization was performed on individual 3 × 3 mm^2^ cells, insulated by mechanical scribing (Micro Diamond MR200 OEG), without a contact grid or antireflective coating.

## Results

### Compositional and Structural Analysis of Sb_2_Se_3_ Nonstoichiometric Thin Films

SLG/Mo/Sb_2_Se_3_ thin films were prepared following the aforementioned experimental methodology. The XRF analysis of the samples revealed that the suggested two-step process allowed us to obtain Sb_2_Se_3_ thin films with the Se amount ranging from 0.36 to 0.55 (2[Se]/(2[Se] + 3[Sb])), where 0.50 corresponds to the stoichiometric conditions. Thus, it was proved that sequential thermal evaporation and reactive annealing synthesis, with control over Se vapor pressure and annealing time, allowed us to incorporate different amounts of Se into the thin films. However, XRF indicated the elemental content in the sample, regardless of whether it forms a single phase or several secondary phases.

**Figure 1:**
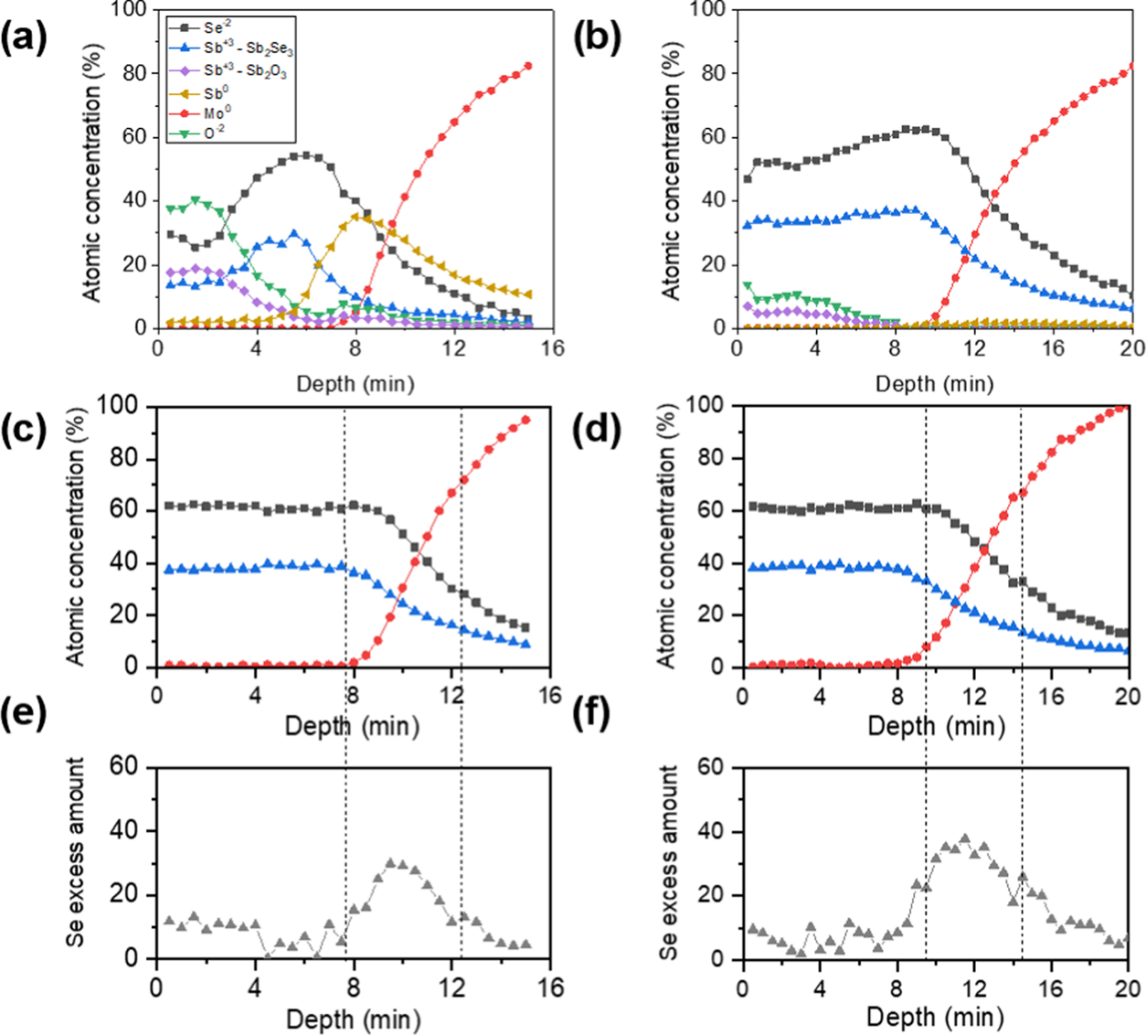
XPS in-depth profiles of samples with a nominal composition of 2[Se]/(2[Se] + 3[Sb]): (a) 0.36, (b) 0.48, (c) 0.53, and (d) 0.54, (e, f) distribution of Se out of the stoichiometry for the 0.53 and 0.54 samples, respectively.

To determine the elemental distribution of the films and to elucidate whether the material admits an extended nonstoichiometric range, in-depth XPS analysis was performed for the different compositions, as shown in Figure 1. In all the cases, it is observed that after a certain sputtering time, the Mo signal increases abruptly, denoting the back Mo/Sb_2_Se_3_ interface. Also, atomic concentrations of Se^–2^ (black spots) and Sb^+3^—in Sb_2_Se_3_—(blue spots) evolve correlatively, indicating that the Sb_2_Se_3_ composition remains steady throughout the film thickness, regardless of the overall Se content as measured by XRF. This suggests that Sb_2_Se_3_ is a single-phase stoichiometric material, whereby any Se or Sb excess in the sample is compensated as secondary phases, rather than forming off-stoichiometry Sb_2_Se_3_. Indeed, for Sb-rich conditions (see Figure 1a,b), an Sb_2_O_3_ phase has been detected at the front of the cell, the amount of which increases as the nominal Se content decreases, indicating that unreacted Sb on the surface of the film has been oxidized following the annealing process, once the material came in contact with air. Interestingly, it is observed that Se has a lower concentration near the surface than in the deeper region of Sb-rich samples (0.36 and 0.48, respectively). A possible hypothesis would be that during the heating ramp, there exists a Se flux toward Mo, before the Sb_2_Se_3_ crystallizes upon reaching its formation temperature around 320 °C. Under Se-deficit conditions, most Se element will have diffused to the back, leading to the aforementioned Sb_2_Se_3_ profile. Recent experiments with interrupted growth processing have shown similar trends; however, more experiments are required to unequivocally confirm the hypothesis.^41^ Finally, Figure 1a shows a high amount of metallic Sb at the back of the absorber, suggesting that the very low Se amount in such a sample is insufficient to react with the Sb precursor throughout its entire thickness.

On the other hand, in the samples with 0.53 and 0.54 Se amounts (see Figure 1c,d), an offset between the Se^–2^ and Sb^+3^ signals, overlapping the Mo curve, is observed. This may indicate that a Se-rich phase has appeared in the back contact interface. To observe more clearly the distribution of Se under these circumstances, the amount of Se in excess (beyond stoichiometry) has been plotted as a function of the sputtering time; see Figure 1e,f. Interestingly, it can be noticed that the Se excess remains fairly low for most of the layer (within the noise range), while significantly increasing in the rear interface. The fact that the amount of Se excess at the back interface increases as the film becomes Se-richer supports the hypothesis that under Se-excess conditions, a Mo–Se phase appears, whose content increases as the overall Se content increases. This phase is likely to be MoSe_2_, as consistently reported for chalcogenide films grown on the Mo substrate.^42−44^

**Figure 2:**
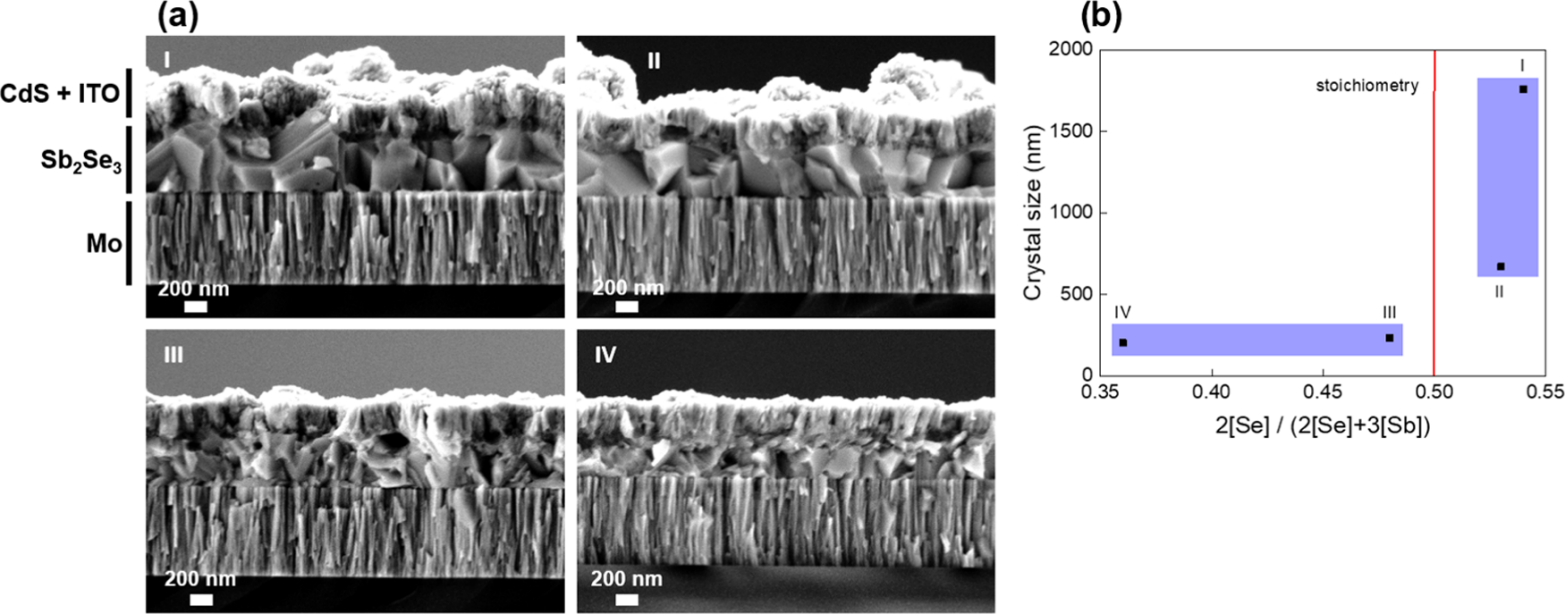
(a) SEM cross-sectional 30× images of samples with 2[Se]/(2[Se] + 3[Sb]): (I) 0.54, (II) 0.53, (III) 0.48, and (IV) 0.36. (b) Crystalline domain as a function of 2[Se]/(2[Se] + 3[Sb]), obtained from the XRD–Rietveld analysis.

To study the structural properties of the Sb_2_Se_3_ phase in either of the previous cases, SEM cross-sectional images of the completed devices are acquired; see Figure 2a. Images have been obtained from complete devices (including buffer and window layers)—see Figure S1 for a complete energy dispersive X-ray analysis (EDX) profile and mapping compositional analysis of the layers constituting a sample. Interestingly, the Se-rich films (I and II) have large grains all developed alongside the entire thickness of the layer, while the Sb-rich samples (III and IV) exhibit smaller grains scattered throughout the thickness of the layer (see Figure S2 for lower magnification images). Moreover, the shape of the grains appears much more random in these samples, whereas I and II show well-oriented grains in the growth direction. To delve deeper into the crystalline orientation and structure of nonstoichiometric Sb_2_Se_3_, a detailed XRD full-profile Rietveld analysis was performed; see Table S1. The average crystal size (Lorentzian) shows a clear threshold around stoichiometric conditions (0.50), from which the crystal size increases significantly as the overall Se amount increases, whereas by shifting toward Sb-rich conditions, it drops abruptly and the slope becomes much less pronounced; see Figure 2b. Importantly, this diverging behavior between Se-rich and Sb-rich conditions clearly implies a change in the growing conditions that favors a more orderly and controlled formation of the grains; one of the goals of this work is to determine the cause. As shown below, this trend is consistently repeated with the other material properties under analysis.

**Figure 3:**
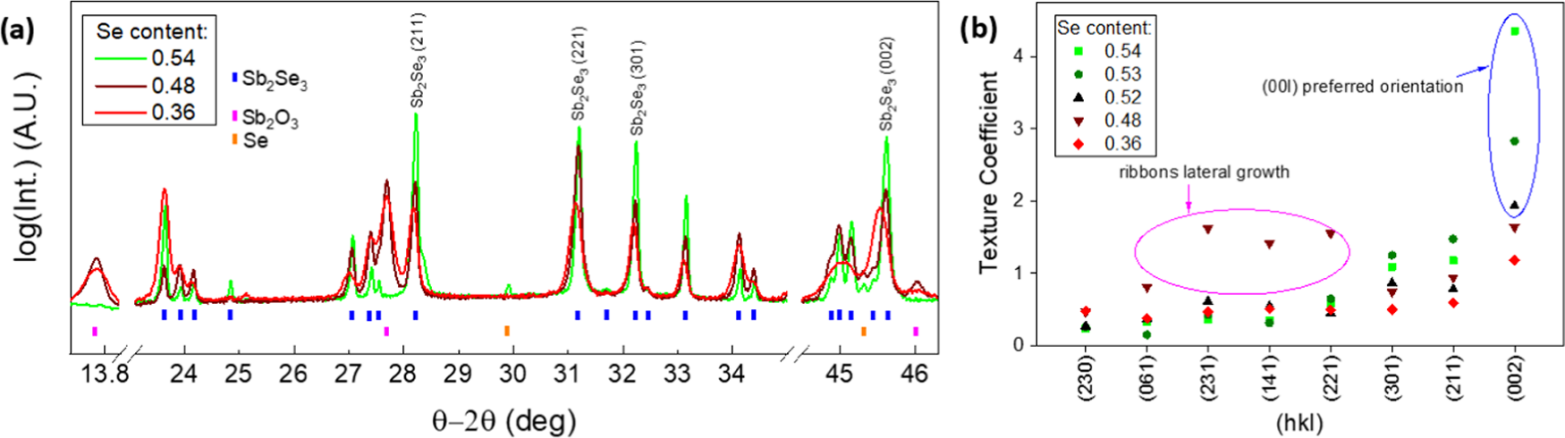
(a) X-ray diffractograms of a set of samples with different 2[Se]/(2[Se] + 3[Sb]) relative amounts. ICDD Powder Diffraction File pattern numbers are Sb_2_O_3_: 01-072-1334 and Sb_2_Se_3_: 04-003-0715. (b) Texture coefficients of diffraction peaks of Sb_2_Se_3_ thin films with different Se amounts.

Figure 3a shows the X-ray diffractograms of samples in each of the compositional ranges of interest for this study, i.e., Sb-rich (2[Se]/(2[Se] + 3[Sb]) = 0.36), slightly Sb-rich/near stoichiometry (0.48), and moderately Se-rich (>0.54), focusing on the selected 2θ ranges; see Figure S3 for the complete 10–140° diffractograms, the Rietveld-calculated patterns, and the difference between the experimental and theoretical patterns. Since the XRD analysis was performed on Mo/Sb_2_Se_3_ samples, the main crystalline phases include Mo (back contact layer) and Sb_2_Se_3_ (absorber). Also, for the Se-rich films, a certain amount of MoSe_2_ and Se secondary phases have been detected, which increase as the overall concentration becomes Se-richer (see Table S1 for main Rietveld refinement analysis parameters including the phase amount, crystal size, and cell volume). In contrast, for Sb-rich thin films, these phases are not observed; however, important amounts of α-Sb_2_O_3_ (0.48, 0.36) and m-Sb (0.36) are detected, whose content also increases as the composition becomes Se-poorer. The presence of the Se and α-Sb_2_O_3_ phases in each group of the samples is directly corroborated from the diffractograms; see Figure 3a. Although with very weak and wide peaks, which are difficult to identify, reflections corresponding to MoSe_2_, mainly oriented in the (001) direction, have been detected and quantified in the Se-rich samples by Rietveld analysis (Table S1); see Figure S4 for the enlarged diffractogram in the 11–15° region, where the MoSe_2_ peak (003) appears.^45,46^ Moreover, a change in the Sb_2_Se_3_ crystallinity depending on the Se content in the films can also be observed. Overall, Sb_2_Se_3_ layers show a complex multiaxial crystallinity, with a [001]-preferred orientation (also in the [*hk*1] and [h01] directions), which is manifested by the intense and sharp peaks observed at 45.6° (002), 29.2° (211), 31.2° (221), and 32.2° (301). Interestingly, as the Se content decreases, these reflections become broader and their intensity declines, until no preferential texture is visibly discerned for the sample with the lowest Se amount. Therefore, it is shown that despite a single-phase stoichiometric material, the crystalline orientation and structure of Sb_2_Se_3_ are indeed affected by the overall Se content of the film, exhibiting a good [001]-preferred orientation for Se-rich samples and deteriorated randomized texture in the Sb-rich range. To quantitatively assess the differences in the crystalline orientation between samples with a distinct Se concentration, the texture coefficient (TC) of the selected Bragg reflections was calculated based on eq 1, where *N* is the number of reflections considered for the calculation, *I*_*hkl*_ is the measured empirical intensity of a diffraction peak, and *I*_0,*hkl*_ corresponds to the intensity value in the standard XRD pattern (ICDD 04-003-0715 Powder Diffraction File patterns).^47,48^
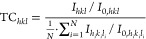
1The results of the TC calculations from a series of selected Sb_2_Se_3_ samples are illustrated in Figure 3b. Notably, samples with Se excess and most specially those with 0.54 Se are primarily dominated by the (002) reflection. Since the *h* and *k* Miller indices are both 0, this indicates that (Sb_4_Se_6_)*_n_* ribbons grow perpendicularly to the substrate surface, proving that Se-rich conditions help enhance the orientation of Sb_2_Se_3_ grains. On the other hand, as we shift toward Sb-rich conditions, the (231), (141), and (221) peaks become dominant. In this case, *h* and *k* values are greater or equal to 3, indicating that the angles between the (Sb_4_Se_6_)*_n_* ribbons and substrate are small, revealing an Sb_2_Se_3_ lateral growth deemed detrimental to the electrical properties of the material.^17^ Finally, we observe that the sample with 0.36 Se has a virtually arbitrary crystalline orientation, with all TC presenting very small, similar values between 0 and 1. However, we note that this sample has the smallest value of TC_002_ and the largest value of TC_230_, indicating a total loss of the preferred orientation in the (002) direction.

In addition, the XRD–Rietveld analysis has showed a connection between the cell volume and the overall Se amount; see Figure S5. First, it is noticed that the cell volume of Sb_2_Se_3_ thin films is smaller than that of the bulk monocrystals.^49^ This reduction of the volume could be associated with a decrease of Se_Sb_ and 2Se_Sb_ defects and an increase of *V*_Se_ vacancies, allegedly reducing the Fermi level splitting (which could affect negatively the *V*_oc_ since Se_Sb_ is responsible for p-type conductivity).^26^ Interestingly, near stoichiometry, this compressive effect is larger, with samples having a cell volume of 543.3 Å^3^ against that of 545.6 Å^3^ of a single crystal, while for the Se(Sb)-richer films, a sequential increase is observed as the nominal composition moves away from stoichiometry, the cell volume becoming more similar to that of the single crystal. It is possible that an increase in the overall Se content compensates the detrimental *V*_Se_ defects, lessening the compressive strain, hence the volume enlargement. However, further research is required to assess the effect of changing the Se amount on the defect structure of Sb_2_Se_3_, in particular at the intrinsic doping level (since otherwise it has been proven that Sb_2_Se_3_ does not admit off-stoichiometry compositions) and how this can affect the cell volume.

**Figure 4:**
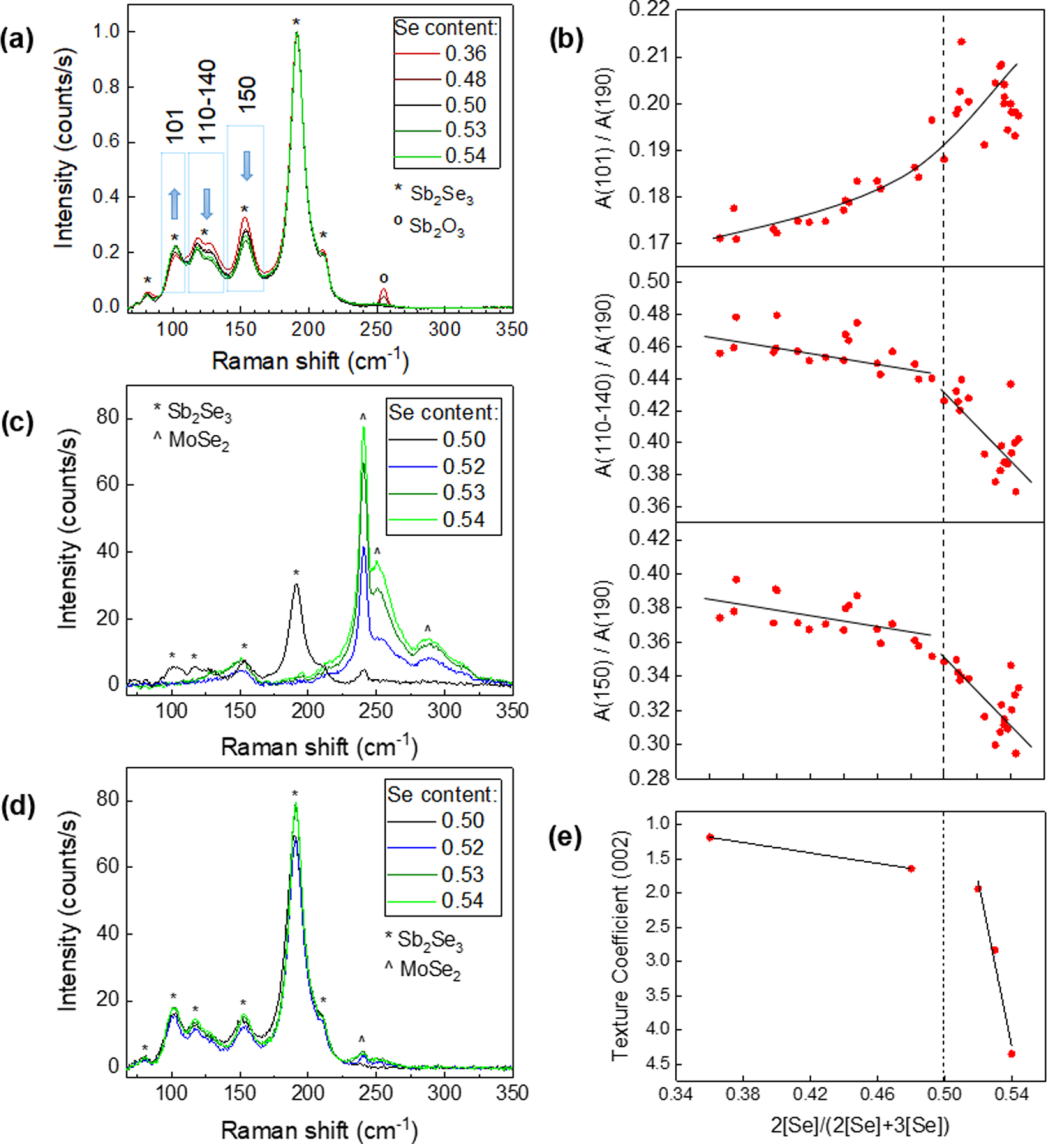
(a) Normalized Raman spectra of the surface of Sb_2_Se_3_ thin films with 2[Se]/(2[Se] + 3[Sb]): 0.36, 0.48, 0.50, 0.53, and 0.54. (b) Area of Raman peaks—expressed as *A*(peak of interest)/*A*(190 cm^–1^) as a function of 2[Se]/(2[Se] + 3[Sb]). (c) Absolute Raman spectra of lift-off remain on the Mo substrate of Sb_2_Se_3_ samples with 2[Se]/(2[Se] + 3[Sb]): 0.50, 0.52, 0.53, and 0.54. (d) Absolute Raman spectra of the previous peeled-off Sb_2_Se_3_ films. (e) Texture coefficient (002) as a function of 2[Se]/(2[Se] + 3[Sb]).

Complementing the previous study of structural properties by XRD and now paying special attention to the characterization of interfaces (both the surface and back interface), a complete Raman spectroscopy analysis was performed. The average Raman spectra of bare Sb_2_Se_3_ thin films (surface) with a nominal composition in the range of 0.36 < 2[Se]/(2[Se] + 3[Sb]) < 0.54 are shown in Figure 4a. The formation of the α-Sb_2_O_3_ secondary phase, which appears only on the surface of Sb-rich samples (peak at 254 cm^–1^), is also detected.^49−51^ On the other hand, elemental Se phases (t-Se)^49,52^ have been detected in the Se-richer samples; see Figure S6. Furthermore, the following trends in some areas of the Raman peaks associated with the Sb_2_Se_3_ phase are also observed (boxed areas in Figure 4a):

1. The area of the Sb_2_Se_3_ peak at 101 cm^–1^ increases as the overall Se amount increases.
2. The area of the Sb_2_Se_3_ peaks between 110 and 140 cm^–1^ decreases as the overall Se amount increases.
3. The area of the Sb_2_Se_3_ peak at 150 cm^–1^ decreases as the overall Se amount increases.

As previously reported by Fleck et al.,^53^ an enhanced ribbon alignment in the [001] direction leads to minimization of the Raman signal at 150 cm^–1^, proving that Raman spectroscopy allows us to quickly and easily assess the quality of the crystalline orientation in Sb_2_Se_3_ thin films. In addition, it is shown here that the areas defined by the 101 and 110–140 cm^–1^ peaks also experience clear variations depending on the 2[Se]/(2[Se] + 3[Sb]) relative concentration of the films increasing and decreasing. The fact that these variations occur in samples where the Sb_2_Se_3_ phase is single-phase stoichiometric, as shown with XPS, but with different crystalline orientations (see Figure 3) leads us to think that the area defined by the 101 and 110–140 cm^–1^ signals is also contingent on the loss of the (002)-preferred orientation, which will be discussed later. The probing depth of the Raman measurements is surface-sensitive (∼100 nm), although their consistency with other methods (XRD), the small thickness of the films (∼800 nm), and the relatively large size of grains (Figure 2) allow us to hypothesize that these observations are also valid throughout the entire film thickness.

Further analysis related to the area variations in Raman spectra and their correlation with the Sb_2_Se_3_ nominal composition of several samples (including samples with a lateral compositional gradient) has been performed; see Figure 4b. Hence, the areas of the 101, 110–140, and 150 cm^–1^ Raman peaks, normalized to the main Sb_2_Se_3_ signal at 190 cm^–1^, have been plotted as a function of 2[Se]/(2[Se] + 3[Sb]). Accordingly, it is clearly noticed that there is a threshold around 0.50 Se, so that for increasing Se amounts, the areas of signals at 150 and 110–140 cm^–1^ stop decreasing at the same rate, and the slope becomes more pronounced. Significantly, these behaviors present similarities with the trends in the crystal size (Figure 2b) and texture coefficient, TC (Figure 4e), where TC_002_ also shows a continuously decreasing slope that becomes more pronounced as the Se amount increases beyond the stoichiometric point. Thus, it is confirmed that both the 110–140 cm^–1^ band and 150 cm^–1^ peak reflect changes in the crystalline orientation, with their areas decreasing as the (002) direction becomes dominant. Otherwise, the 101 cm^–1^ signal presents a similar behavior with a positive slope, which is consistent with the opposite symmetry of this peak. The trend may not be as clear as that for the 110–140 and 150 cm^–1^ signals; however, the area of integration is also smaller, leading to the signal/noise ratio of the measurement affecting more pronouncedly the dispersion of data points.

These observations raise a question: if Sb_2_Se_3_ is indeed a stoichiometric material, why does it have an enhanced ribbon alignment when the overall composition is Se-rich? To delve deeper into this behavior, a mechanical lift-off of a series of samples with different 2[Se]/(2[Se] + 3[Sb]) nominal concentrations was performed. See Figure 4c for the lift-off remains on the Mo substrate for samples with the Se concentration ranging from 0.50 to 0.54. Interestingly, a MoSe_2_ phase has been identified, whose content increases as the overall Se amount also increases, while for the stoichiometric sample (0.50), Sb_2_Se_3_ is observed and only traces of MoSe_2_ are detected. Note that this trend coincides with the loss of the (002)-preferred orientation as shown by XRD and Raman. Thus, it is likely that the *in situ* formation of MoSe_2_ plays a crucial role in the growth of well-oriented Sb_2_Se_3_. Likewise, the absence of MoSe_2_ leads to a more randomized grain growth, without the optimal ribbon alignment perpendicular to the substrate, which is likely to negatively affect the device performance due to lower conductivity across the parallel-stacked ribbons. This has been further validated by preparing an SLG/Sb_2_Se_3_ sample following identical synthesis conditions to those for manufacturing an SLG/Mo/Sb_2_Se_3_ (0.54 Se) film and performing the corresponding XRD analysis; see the diffractogram in Figure S7 and calculated TCs in Table S2. The TC_002_ of an SLG/Sb_2_Se_3_ sample (1.8) is significantly lower than that of Se-rich SLG/Mo/Sb_2_Se_3_ (4.4). Moreover, TC_230_ is larger (0.8 against 0.2 of SLG/Mo/Sb_2_Se_3_), showing that films directly grown on glass do not present a (002)-preferred crystalline orientation. Finally, additional Raman measurements are performed on the peeled-off Sb_2_Se_3_ film (see Figure 4d), where the expected Sb_2_Se_3_ signals can be easily noticed, along with some of MoSe_2_, whose amount increases as the film becomes Se-richer.

Here, it has been demonstrated that an enhanced Sb_2_Se_3_ ribbon alignment leads to both a decline in the area of 150 and 110–140 cm^–1^ Raman peaks and an increase of the 101 cm^–1^ signal, deeming Raman spectroscopy as a powerful technique to assess how well-oriented the material is. Additionally, two discrepant trends have been reported based on the Se nominal composition, denoting that variations in the crystalline orientation, as shown in Figure 4b,e, must originate from two combined effects: first, the increasing annealing times between samples 0.36 and 0.50 (15–30 min), leading to a progressive and soft enhancement of the preferred [hk1] orientation until stoichiometry is reached (slope of 0.2 cm^–1^ in Figure 4b, 110–140 and 150 cm^–1^ peaks); and second, the augmenting content of MoSe_2_ in samples 0.50–0.55, producing an abrupt change of slope (1 cm^–1^ in Figure 4b) and ribbon alignment, leading to fully oriented grains in the (001) direction. These observations confirm previous accounts by Li et al.,^48^ where selenylation of Mo prior to the Sb_2_Se_3_ deposition led to an enhancement of [*kh*1]-oriented grains. The fact that MoSe_2_ has a preferred crystalline orientation in the (001) direction (see Rietveld analysis in Table S1) suggests that the preferential perpendicular growth of MoSe_2_ favors the vertical anchoring of (Sb_4_Se_6_)*_n_* ribbons, forming oriented Sb_2_Se_3_ grains in the (002) direction.^43,54^ Therefore, modifying the texture of the back contact (from Mo to the Mo/MoSe_2_ system) ultimately affects the formation of Sb_2_Se_3_ by enhancing its selectively oriented growth in the (001) direction, essential to develop a good quasi-1D structure. This is supported by previous research, according to which the substrate affects all electronic, structural, and crystallographic properties of the absorber.^18,19^

Regarding the role of MoSe_2_, we can point to two factors that lend weight to our hypothesis about its effect on the oriented growth of Sb_2_Se_3_. First, since the enthalpy of formation of MoSe_2_ (− 234.4 kJ/mol) is negative and smaller than that of Sb_2_Se_3_ (−128.7 kJ/mol), i.e., MoSe_2_ is thermodynamically favored under our manufacturing conditions; and experimental evidence indicates that there is a Se flux toward Mo during the heating process (see Figure 1), it is plausible to assume that the MoSe_2_ layer is formed in the first place, effectively influencing the subsequent Sb_2_Se_3_ crystallization.^55^ Second, the lattice mismatch (see eq 2, where ε stands for the mismatch and *a*_*i*_ is the lattice parameter of each layer) of the MoSe_2_/Sb_2_Se_3_ interface is significantly smaller (8%) than that of Mo/Sb_2_Se_3_ (26%) when both the substrate and layer are oriented in the *c* direction; see the lattice constants and mismatch in Table S3.^56−58^ This indicates that the strain between MoSe_2_ and Sb_2_Se_3_ layers with the preferred crystalline orientation is lower than that of the Mo/Sb_2_Se_3_ system, which may contribute to a more ordered ribbon growth in the first case. Also, a mismatch larger than 20% inevitably implies the formation of incoherent interfaces, while for ε < 10%, the system may be strained but leads to coherent interfaces.^59^
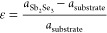
2To sum up, the previous compositional and structural analysis of Sb_2_Se_3_ nonstoichiometric thin films has led to the following observations:

- Sb_2_Se_3_ is a single-phase compound that does not allow an extended range of nonstoichiometry conditions. Indeed, any Sb or Se excess is compensated in the form of secondary phases: Sb_2_O_3_ and m-Sb for Sb-rich conditions and MoSe_2_ and amorphous Se for Se-rich conditions.
- Under Sb-rich conditions, the crystal domain is small and grains grow in a disordered manner. In Se-rich films, grains are as thick as the layer itself with only grain boundaries parallel to device thickness.
- The crystalline orientation and structure of Sb_2_Se_3_ films are affected by the nominal Se content. A clear tendency toward an improved (002) orientation is maintained until stoichiometry is reached (most likely related to the increased annealing times and fewer impurity phases when approaching the 0.50 threshold). In contrast, for the increased Se content, the presence of MoSe_2_ in the rear contact of the films plays a crucial role in the growth of well-oriented polycrystalline layers, leading to an abrupt enhancement of the (002) reflections.

### Absorption Coefficient

Finally, photothermal deflection spectroscopy (PDS) was used to study the effect of varying the overall Se amount on the optical band gap and Urbach energy of Glass/Sb_2_Se_3_ samples. This information is very relevant since it allows us to assess the degree up to which changes in the nominal composition can affect intrinsic properties of the material, such as band gap energy. Also, exponentially decaying density of state (DOS) tails due to low-quality crystalline materials may cause transitions between these energy states inside the band gap (Urbach tails), which are characterized by the Urbach energy (*U*_0_), leading to sub-band gap absorption. It is known that *U*_0_ has an impact on carrier mobility and lifetime, affecting the device performance, especially the open-circuit voltage.^60,61^ Hence, by computing *U*_0_, it is possible to characterize the influence of modifying the overall Se content on the performance of the cell, with regard to the presence of band tails. Also, considering that these observations relate only to the absorber rather than the complete junction, they allow us to decorrelate the limitations from the films and the limitations from a specific junction, such as interfacial intermixing and band alignment, offering a good approach to characterize the defects of the material itself.

**Figure 5:**
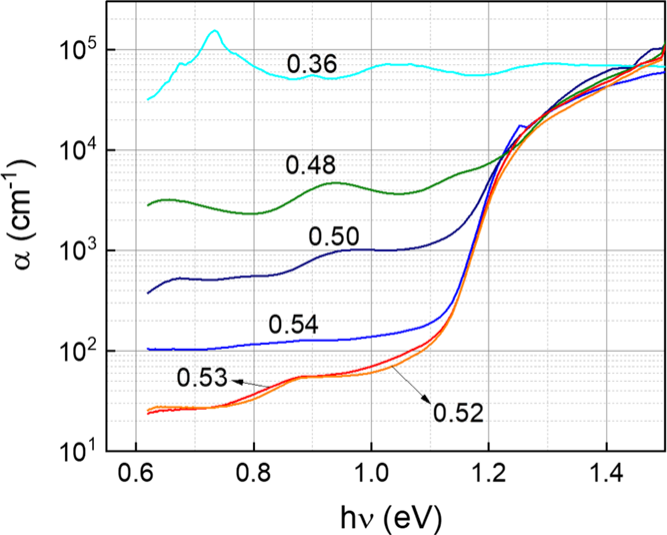
Absorption coefficient (α) as a function of photon energy (*h*ν) of glass/Sb_2_Se_3_ films prepared during the same synthesis process as the SLG/Mo/Sb_2_Se_3_ corresponding samples with 2[Se]/(2[Se] + 3[Sb]): 0.54, 0.53, 0.50, 0.48, and 0.36. The absorption coefficient obtained from the PDS spectra.

The absorption coefficient α is represented as a function of the energy of the incident (exciting) beam in Figure 5a. It is clear that the sub-band gap absorption increases very significantly as the overall Se amount decreases. For instance, the sample with 0.36 Se (2[Se]/(2[Se] + 3[Sb])) does not even show a band gap decay, which could result from the large number of secondary phases (see XPS results, Figure 2), masking the Sb_2_Se_3_ absorption and leading to a very high α below the Sb_2_Se_3_ band gap (in the order of 10^5^ cm^–1^). Otherwise, improvements when switching from the stoichiometry (0.50) toward Se-rich conditions (0.52, 0.53) are noticed—the absorption coefficient inside the band gap decreases from 10^3^ to 10^1^ cm^–1^ (2 orders of magnitude). In the absence of a more detailed study on the effects of intrinsic doping for a very small interval around the stoichiometry, this significant decrease in the sub-band gap absorption may be due to compensation of deep defects. Interestingly, the samples with the lowest subgap α are those with 0.52 and 0.53 Se, whereas those with 0.54 are slightly higher, suggesting that a large Se excess can be detrimental to the absorber, possibly due to the appearance of Se secondary phases on the surface (see Raman spectra with elemental Se peaks in Figure S6). The effect of Se phases in the front interface could be assessed by comparing as-grown samples with films subjected to selective or complete surface etching (Br_2_).^62−64^ Other than that, the larger subgap absorption in Se-richer thin films could also be due to the recently reported abnormal defect behavior in Sb_2_Se_3_, which causes the density of Se vacancies *V*_Se_ to increase as the material becomes anion-richer, leading to general deteriorated performance in extremely Se-rich films.^65^

Tauc plots were computed from the PDS-derived α, from which the value of the optical band gap was extrapolated; see the obtained values in Table 1. Note that all values are comparable considering the uncertainty. This confirms our hypothesis that Sb_2_Se_3_ is a stoichiometric material. Even though different nominal compositions have been explored, the band gap remains unchanged, indicating that no compositional changes of the Sb_2_Se_3_ phase are expected. Finally, the Urbach energy is determined in accordance with eq 3, where α_0_ and U_1_ are constants determined by fits to the experiment, *h* is Planck’s constant, and *v* is the incident beam frequency. Therefore, *U*_0_ is obtained from the inverse of the slope resulting from plotting ln(α) as a function of energy; see results in Table 1.^17^ In the Se-rich range, *U*_0_ is low (23–24 meV), indicating a good-quality absorber with a small number of absorption centers inside the band gap. Significantly, the Urbach energy in the Se-rich range is lower than other reported *U*_0_ values from Sb_2_Se_3_-evaporated films.^17,66^ Nonetheless, values shown here are still above the 20 meV barrier, which has been indicated to be one of the factors contributing to the large *V*_oc_ deficit in CIGS and CZTSSe technologies.^60^ On the other hand, when the overall Se amount decreases, the Urbach energy increases steadily. As stated above, all previous measurements point toward a stoichiometric Sb_2_Se_3_ layer regardless of the synthesis conditions. Hence, variations in *U*_0_ are likely due to increased disorder of the material, resulting from nonoptimal growth conditions, such as the presence of Sb_2_O_3_ and Sb preventing good crystalline growth (small, randomly oriented grains under Sb-rich conditions; see Figure 2). Changes in Urbarch energy can also be connected to modifications in the defect structure of the material, where deep defects are compensated under Se-rich conditions but are prominent under Sb-rich conditions, all included inside the Sb_2_Se_3_ doping level. Considering both the very low sub-band gap absorption and *U*_0_ of Se-rich Sb_2_Se_3_ thin films, it is expected that these can lead to better performing devices.
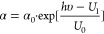
3

**Table 1:** Optical Band Gap (eV) and Urbach Energy (meV) in Relation to the Nominal Se Content in the Samples

Se content	band gap (eV)	Urbach energy (meV)
0.48	1.153 ± 0.022	70.12 ± 0.91
0.50	1.150 ± 0.027	41.80 ± 1.20
0.52	1.144 ± 0.012	24.80 ± 0.37
0.53	1.151 ± 0.008	23.49 ± 0.34
0.54	1.154 ± 0.006	22.99 ± 0.32

### Discussion of Structural and Optical Characterization and Its Translation to the Optoelectronic Properties of Devices

**Figure 6:**
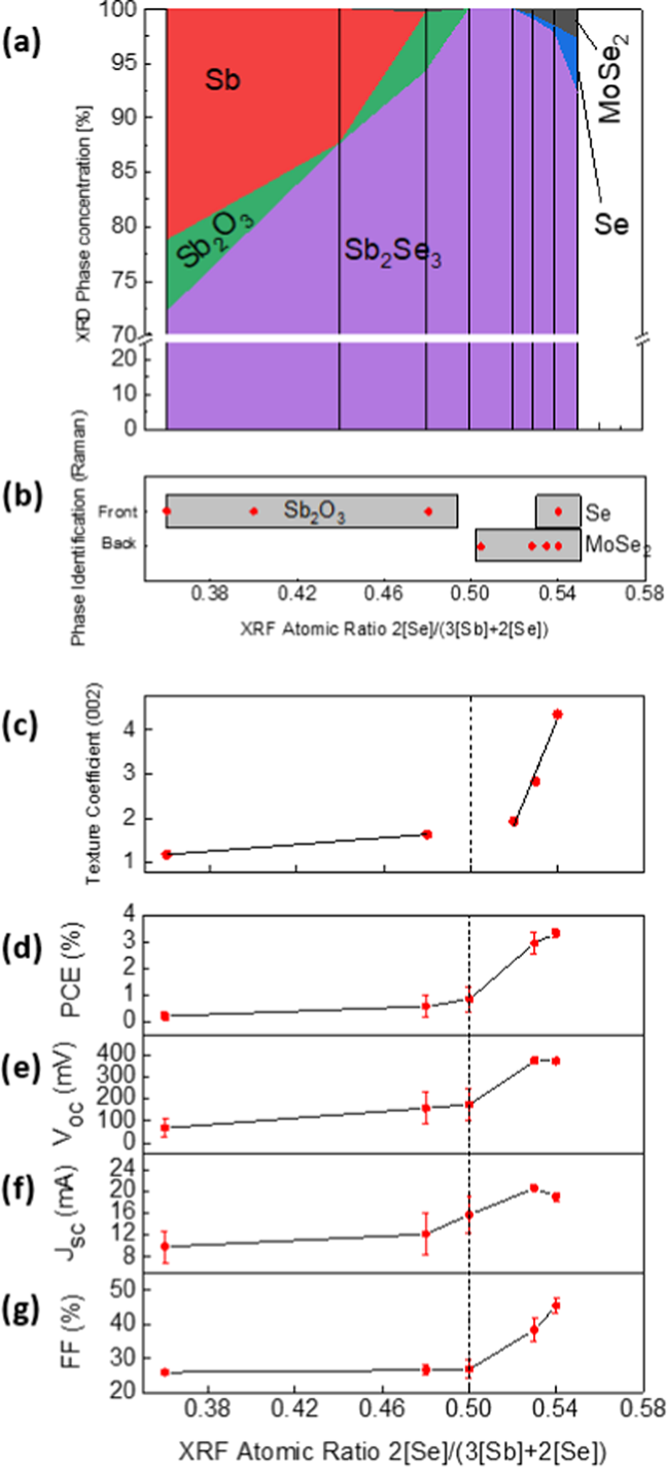
(a) Phase concentration extracted from XRD as a function of 2[Se]/(2[Se] + 3[Sb]). (b) Phases identified by Raman spectroscopy analysis as a function of 2[Se]/(2[Se] + 3[Sb]). (c) Texture coefficient (002) as a function of 2[Se]/(2[Se] + 3[Sb]). (d–g) Evolution of the optoelectronic parameters as a function of 2[Se]/(2[Se] + 3[Sb]).

With the results of the compositional analysis presented above, a phase diagram has been developed; see Figure 6. Accordingly, note that the XRD analysis (Figure 6a) shows different secondary phases depending on whether the material is under Se-rich or Sb-rich conditions, suggesting that Sb_2_Se_3_ does not allow an extended off-stoichiometry range. Instead, Sb excesses are either converted into Sb_2_O_3_ or remain as unreacted m-Sb, whereas Se excesses diffuse toward the Mo back contact, forming a MoSe_2_ layer. Nevertheless, XRD tends to underestimate the MoSe_2_ content, which has been detected in all Se-rich films analyzed by Raman spectroscopy (Figure 6b), demonstrating that MoSe_2_ appears systematically in the Mo/absorber interface of Se-rich Sb_2_Se_3_. Therefore, combining XRD and Raman, it can be inferred that Sb_2_Se_3_ is a stoichiometric material, highly resilient to compositional changes, with any Sb or Se excesses compensated in the form of metallic Sb, Sb_2_O_3_ (Sb-rich), or MoSe_2_ (Se-rich). Moving further away from the stoichiometry toward Sb-rich conditions, the amount of unreacted Sb increases. On the other hand, for 2[Se]/(2[Se] + 3[Sb]) > 0.54, Se elemental phases appear, which could likely be a limitation for electronic transport.

So far, the effect of varying the overall Se concentration in Sb_2_Se_3_ on the intrinsic properties of the absorber (structure, composition, morphology) has been considered, regardless of how it might affect its PV performance. To investigate the optoelectronic properties of nonstoichiometric Sb_2_Se_3_-based solar cells, *J*–*V* curves of a selected set of samples have been acquired, which are processed into PV devices with the following architecture: Mo/Sb_2_Se_3_/CdS/ZnO/ITO. The results from this study are shown in Figure 6d–g, where the power conversion efficiency (PCE), *V*_oc_, *J*_sc_, and fill factor (FF) have been plotted as a function of 2[Se]/(2[Se] + 3[Sb]). Notice that PCE and *V*_oc_ follow similar trends (Figure 6d,e), with the devices prepared from absorbers with 0.36 and 0.48 Se showing the lowest *V*_oc_ (66 and 157 mV, respectively), which could be due to the abundant secondary phases appearing under Sb-rich conditions, the high Urbach energy (see Table 1), and the extremely large sub-band gap absorption (see PDS results in Figure 5a). These secondary phases include an Sb_2_O_3_ layer in front of the cell (see XPS profiles in Figure 1), thicker than a few nanometers, and possibly inhomogeneous morphology is likely to disrupt the Sb_2_Se_3_/CdS interface, preventing a good performance of the p-n junction. On the other hand, both 0.53 and 0.54 Se films exhibit a good PCE and *V*_oc_ of around 4% and 400 mV, respectively, with which it is shown that a small deviation toward Se-poor compositions is much more detrimental to the operation of the device than switching toward Se-rich conditions. However, as the Se amount continues to increase, a small reduction in *J*_sc_ is observed (see Figure 6f), which could result from the appearance of Se elemental phases at the heterointerface with CdS (Figure S6).

More interestingly, the sample with 0.50 Se (strictly stoichiometric) shows low PCE, *V*_oc_, and FF, when under these conditions of absence of impurity phases, much higher values, or at least similar to those of the 0.53 and 0.54 films (Se-rich), would be expected. On the one hand, the better performance of Se-rich films could be due to the compensation of *V*_Se_ defects, which might act as effective recombination centers.^26^ On the other hand, the reported declining trends in *V*_oc_ and FF coincide with the loss of a (002)-preferred crystalline orientation when the Se content decreases, as shown by TC_002_ in Figure 6c. Indeed, all optoelectronic parameters in the range of 0.36 < 2[Se]/(2[Se] + 3[Sb]) < 0.54 present the characteristic double-slope trend of TC_002_, indicating a strong dependence of Sb_2_Se_3_ PV performance on the crystalline orientation. A deterioration in the ribbon alignment implies the loss of the enhanced *c*-axis conductivity due to the anisotropic properties of Sb_2_Se_3_, causing a sharp drop in the PV performance.

Furthermore, it is observed that while the MoSe_2_ content gradually decreases until it stops forming for Sb-rich samples, TC_002_ decreases steeply to values between 1 and 2. Afterward, the (002)-preferred orientation continues dwindling, but with a much lower rate. Therefore, it is likely that the existence of a MoSe_2_ phase plays a relevant role in the well-oriented growth of quasi-1D Sb_2_Se_3_. Thus, a certain amount and good crystalline orientation of MoSe_2_ can decisively influence the preferential orientation of Sb_2_Se_3_, improving its optoelectronic properties. Certainly, by the XRD–Rietveld analysis, it has been noticed that the MoSe_2_ has a preferred crystalline orientation in the (001) direction, confirming its role in enhancing the Sb_2_Se_3_ grain growth perpendicular to the substrate (see Table S1).

Overall, these results clearly point out in the direction that Sb_2_Se_3_ is a single-phase material that does not admit significant off-stoichiometric variations, whose crystalline orientation is essential to develop good PV performance, and for which the growing conditions, in particular with regard to the formation of certain phases, can either improve or deteriorate its structural and electronic properties. However, the study presented here has some limitations. For instance, although it confirms previously reported improvements of incorporating a MoSe_2_ layer in Mo/Sb_2_Se_3_ devices^48^ (in this case by *in situ* formation of MoSe_2_ during the Sb_2_Se_3_ reactive annealing), no specific analysis of the MoSe_2_ phase has been performed. Thus, we believe that studying the formation process and properties of the MoSe_2_ phase in detail could help to clear up some of the hypotheses raised in this work, such as the specific effect it exerts on the Sb_2_Se_3_ crystalline growth. Furthermore, results suggest that the Sb_2_Se_3_ formation by reactive annealing constitutes a dynamic process, in which there is a Se flux through the Sb layer, and then, the absorber crystallizes upon reaching 320 °C, validating the premise that the back contact’s texture has an impact on the Sb_2_Se_3_ structure. However, the precise formation mechanism should be investigated further—we suggest performing complete characterization analysis of samples acquired by interrupted growth at different annealing times.^41^ Also, this work is essentially centered on the standard process for preparing Sb_2_Se_3_ thin films in the substrate configuration (i.e., Mo/Sb_2_Se_3_/buffer layer/TCO), leading to the formation of the MoSe_2_ layer under Se-excess conditions. Notwithstanding, we believe that it might be interesting to investigate what happens when there is no Mo resource available. Under these circumstances, it may be possible that the Se surplus is effectively incorporated into the Sb_2_Se_3_ structure or else it accumulates at the front interface in the form of elemental Se, as has been suggested elsewhere.^67−69^ Further research and supporting evidence for verification are required.

In this work, we have shown that there is a direct correlation between Raman spectra and (002) crystalline orientation, which so far has been justified based on a comparative study of Raman analysis and XRD (especially TC calculations); however, additional support is required, for example, by studying the Raman spectra of Sb_2_Se_3_ grown on different substrates or using synthetic routes that give rise to different textures. Otherwise, further study of Sb_2_Se_3_ in the Se-rich range might be necessary to unequivocally identify the nature of the elemental Se phases that appear under these conditions and hence design strategies to remove or minimize them, such as etching procedures. Finally, we emphasize that this study has been based on investigating the consequences of significantly modifying the nominal concentration of Sb_2_Se_3_ films; however, focusing on a very small interval around the stoichiometry might shed new light on the effect of intrinsic doping, especially with regard to reducing the amount of *V*_Se_ defects.

## Conclusions

In this work, it has been confirmed that Mo/Sb_2_Se_3_ thin films do not allow an extended off-stoichiometry range, whereby any Sb or Se excess is compensated in the form of secondary phases: MoSe_2_ and Se elemental phases under Se-rich compositional conditions and Sb_2_O_3_ and m-Sb in the Sb-rich range. The results presented here illustrate the difficulty of selectively controlling or enhancing the formation of Sb_2_Se_3_ intrinsic properties (e.g, defects) by modulating the Se chemical potential beyond stoichiometric conditions, unlike CIGS or CZTS. Hence, we suggest that enhancing the many virtues of Sb_2_Se_3_ (and minimizing its numerous defects) will require exploring new opportunities through extrinsic doping or alloying. For instance, the similar behavior of Sb_2_Se_3_ to that of CdTe makes us think about the suitability of applying successful recipes in CdTe to unlock the full potential of Sb_2_Se_3_. Second, it has been shown that despite the fact that Sb_2_Se_3_ is stoichiometric, it is necessary to operate under Se-rich conditions to develop good devices, primarily to form a MoSe_2_ phase — promoting a well-oriented growth in the (002)-crystalline direction and avoiding the appearance of unwanted secondary phases — and also to minimize the number of detrimental *V*_Se_-type defects, although this aspect needs to be investigated further. We demonstrate that MoSe_2_ plays a dual role, as a hole transport layer and as a growth matrix, directing and effectively influencing the structural characteristics of Sb_2_Se_3_.^70^ Ultimately, this study has shown the power of Raman–XRD-related combined techniques to examine the structural properties of Sb_2_Se_3_, especially the degree of orientation in the (001) direction, and PDS to obtain the sub-band gap absorption and *U*_0_, which need to be minimized to develop good absorbers. Overall, this work points out at new strategies to improve Sb_2_Se_3_ devices, including extrinsic doping approaches rather than intrinsic doping (due to the small gap free of damaging secondary phases when the composition shifts away from stoichiometry) and etching treatments for Se-rich samples (to take advantage of the MoSe_2_ formation, while eliminating the surface elemental Se disruptive phases).

## Supporting Information Available

Description of the procedure to obtain the absorption coefficient by PDS; additional XPS, XRD Raman, and SEM figures; and XRD analysis and Rietveld parameters (PDF)
