# Supplementary material for: Does Sb2Se3 Admit Nonstoichiometric Conditions? How Modifying the Overall Se Content Affects the Structural, Optical, and Optoelectronic Properties of Sb2Se3 Thin Films
Source: ACS Appl Mater Interfaces. 2022 Mar 1;14(9):11222–34. doi: 10.1021/acsami.1c20764 (PMC8915164; doi:10.1021/acsami.1c20764)
Supplement: Supplementary file 1 — am1c20764_si_001.pdf [file am1c20764_si_001.pdf]

Supporting Information

Does Sb_2Se_3 admit non-stoichiometric conditions? – How modifying the overall Se content affects structural, optical and optoelectronic properties of Sb_2Se_3 thin films

Ivan Caño[1], Pedro Vidal-Fuentes[2], Lorenzo Calvo-Barrio[3,4], Xavier Alcobé[3], José Miguel Asensi[5], Sergio Giraldo[2], Yudania Sánchez[2], Zacharie Jehl[1], Marcel Placidi[1], Joaquim Puigdollers[1], Victor Izquierdo-Roca[2], Edgardo Saucedo[1]*

[1] Escola d'Enginyeria de Barcelona Est (EEBE), Universitat Politècnica de Catalunya, Av. Eduard Maristany, 16, 08019 Barcelona, Spain

[2] Institut de Recerca en Energia de Catalunya (IREC), Jardins de les Dones de Negre, 1, 08930 Sant Adrià del Besòs, Spain

[3] Centres Científics i Tecnològics (CCiTUB), Universitat de Barcelona, C. Lluis Solé i Sabaris 1-3, 08028 Barcelona, Spain

[4] IN2UB, Departament d'Enginyeria Electrònica i Biomèdica, Universitat de Barcelona, C. Martí i Franquès, 1, 08028 Barcelona, Spain

[5] Departament de Física Aplicada, Universitat de Barcelona, C. Martí i Franquès, 1, 08028 Barcelona, Spain

*Corresponding Author. E-mail: ivan.cano.prades@upc.edu

PDS

PDS is a highly sensitive technique based on the deflection of a laser beam probing a thin layer adjacent to the sample, which is heated with an optically exciting beam, causing a corresponding change in the index of refraction (∇n)[1,2]. A.C. Boccara et al. showed that the amplitude of the probe beam deflection carries information on the optical absorption and thermophysical properties of the sample[3]. Therefore, from the deflection angle it is possible to determine the absorptance of semiconductor thin films, even in ultralow absorption regimes. Whereas in common transmittance and reflectance measurements (from which $Absorptance = 1 - T - R$), low values of absorptance cannot be reliably measured, since the sum of T and R would be close to one, leading to large vagueness in the calculation of absorptance; in PDS only the absorptance A (the part which turns into heat when the sample is irradiated with an exciting pump) is directly measured and therefore, it has great sensitivity for low absorption materials, and especially in the sub-bandgap region. This allows to investigate the presence of absorption centres whose energy is lower than the bandgap, which could be indicative of deep defects and mid-gap states that contribute to carrier recombination, leading to reduced carrier lifetime. Also, from the exponential band-edge decay it is possible to derive the Urbach energy (band tails, intrinsic disorder) and the optical bandgap of the material.

With PDS, we determined the absorptance of a series of Sb_2Se_3 thin films. Then, absorption coefficient α was derived with **Equation S1**; where d is the thickness of the layer (measured by XRF), An is the absorptance (defined as the fraction of non-reflected radiation that is absorbed into the film, measured by PDS), and R is the reflectance of the layer / air interface (defined as the average of the optically measured reflectance in the low-absorption range). This simple

approximation for α is deduced from a layer without substrate ("single slab"), considering the internal reflections by means of R.

$$\alpha = -\frac{1}{d}\ln\left(\frac{1 - An}{1 - R \cdot An}\right)$$

Equation S1

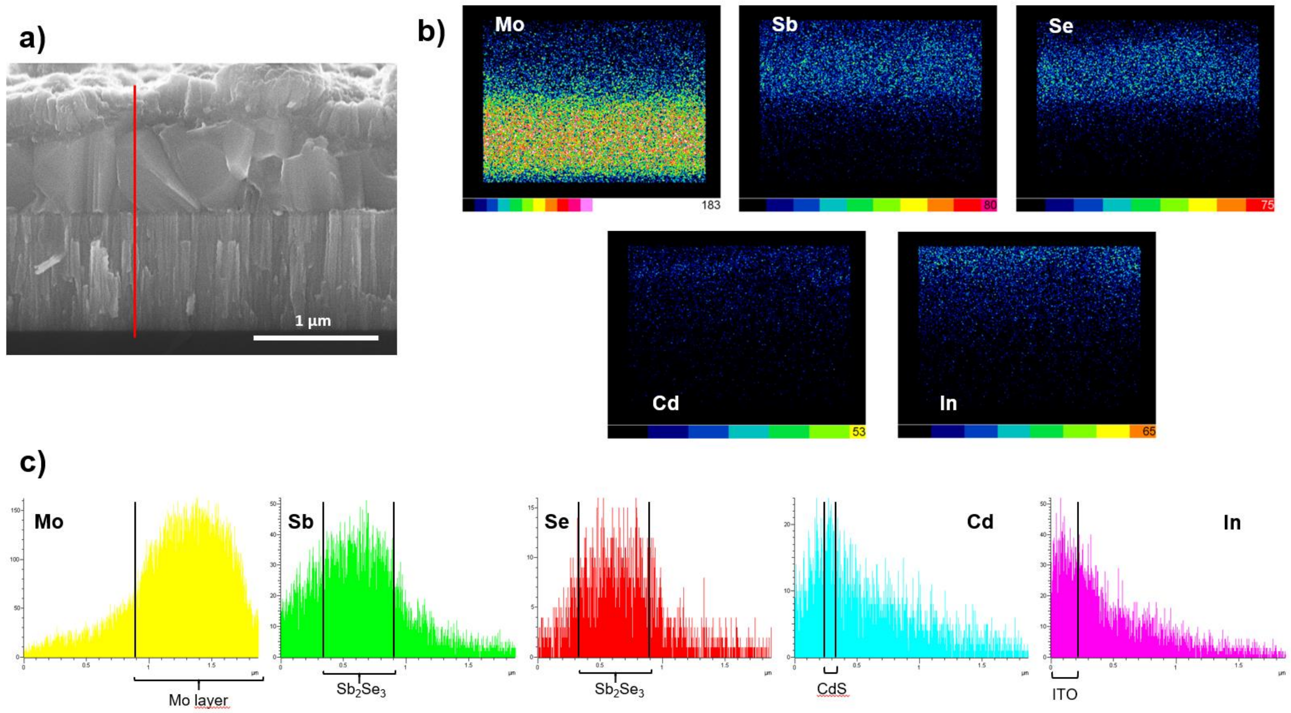

Figure S1: EDX analysis. **a)** 30X SEM image of a Sb_2Se_3 sample with 2[Se]/(2[Se] + 3[Sb]) = 0.54 – red line indicates the EDX profile mapping zone. **b)** EDX zone mapping. **c)** EDX line profile mapping. Note that Mo corresponds to the bottom layer from **Figure 2**a, Sb and Se (Sb_2Se_3) accumulate mainly in the centre of the sample and correspond to the central layer, Cd (from CdS) lies in a thin layer on top of the previous one, and ITO (with In) constitutes the outermost layer

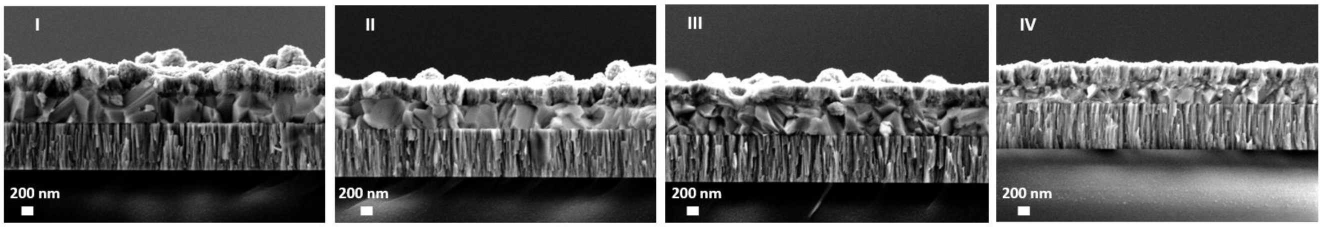

Figure S2: SEM cross-section 20X images of samples with 2[Se]/(2[Se] + 3[Sb]): I) 0.54, II) 0.53, III) 0.48, IV) 0.36

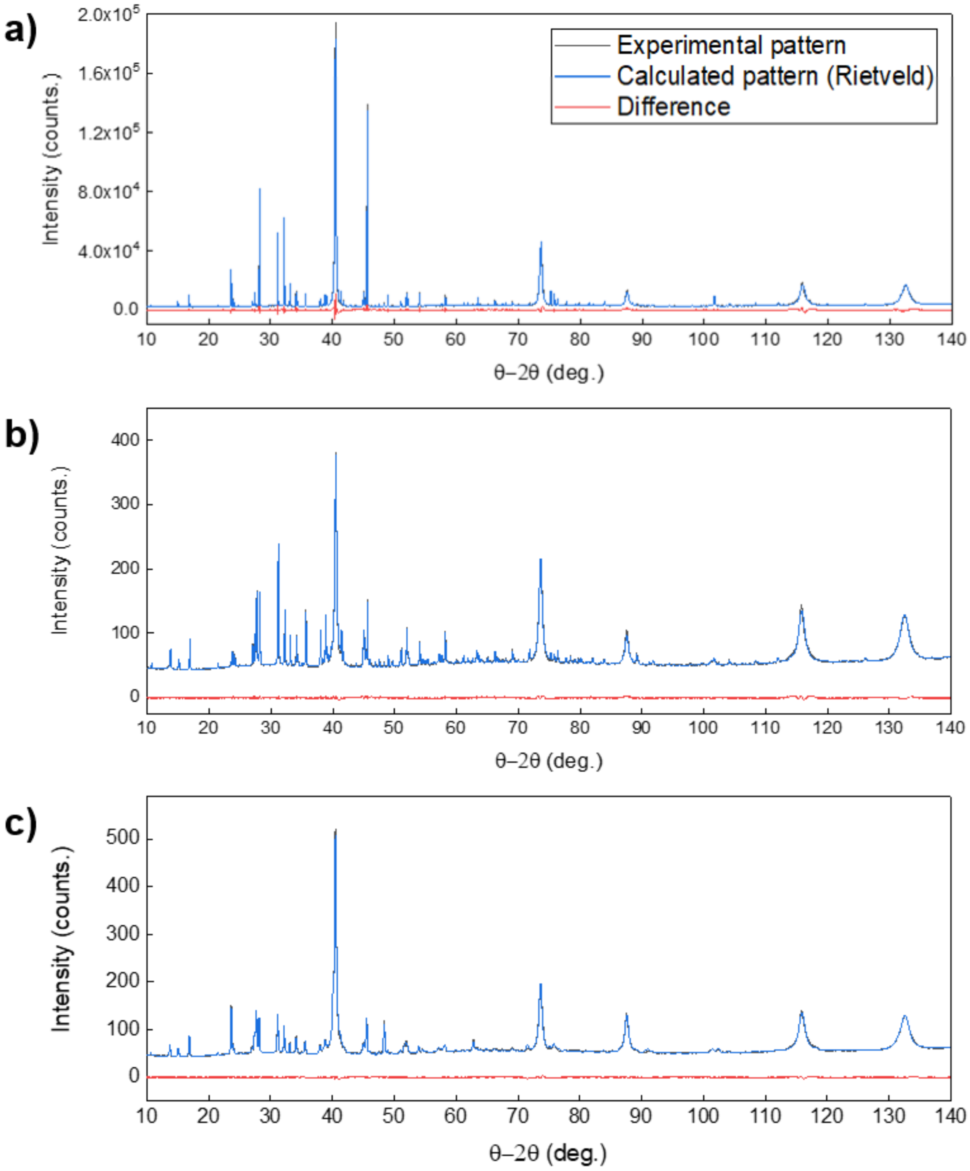

Figure S3: Rietveld refinement (blue) of XRD experimental patterns (black), and difference between observed and theoretical diffractograms (red), of Sb_2Se_3 thin films with 2[Se]/(2[Se] + 3[Sb]): **a)** 0.36. **b)** 0.48. **c)** 0.54

Table S1: Rietveld parameters of Sb_2Se_3 thin films with 2[Se]/(2[Se] + 3[Sb]): 0.54, 0.53, 0.48, 0.44 and 0.36

2[Se]/(2[Se] + 3[Sb])	Phase	Properties of each phase
0.54	Sb_2Se_3	Quantity: 49.433%<br>R-Bragg: 2.030<br>Spacegroup: Pbnm<br>Cell volume: 543.88368 Å^3<br>Crystal size (Lorentzian): 1761.608 nm<br>Strain e0: 0.00013<br>Lattice parameters:<br>a = 11.6270128 Å<br>b = 11.7674458 Å<br>c = 3.9751704 Å
	Mo (substrate)	Quantity: 49.489%<br>R-Bragg: 0.937<br>Spacegroup: Im-3m<br>Lattice parameters:<br>a = 3.1485750 Å
	$MoSe_2$	Quantity: 0.769%<br>R-Bragg: 1.573<br>Spacegroup: P63/mmc<br>Cell volume: 122.60322 Å^3<br>Crystal size (Lorentzian): 9.549 nm<br>Preferred orientation (Dir: 001): 0.7663745<br>Lattice parameters:

		a = 3.3 Å<br>c = 13 Å
	Se	Quantity: 0.309%<br>R-Bragg: 0.983<br>Spacegroup: Pm-3m<br>Preferred orientation (Dir: 110): 0.4165669<br>Lattice parameters:<br>a = 2.9784934 Å
0.53	Sb_2Se_3	Quantity: 37.658%<br>R-Bragg: 2.333<br>Spacegroup: Pbnm<br>Cell volume: 543.31396 Å^3<br>Crystal size (Lorentzian): 607.430 nm<br>Strain e0: 0.00007<br>Lattice parameters:<br>a = 11.6235683 Å<br>b = 11.7626724 Å<br>c = 3.9737944 Å
	Mo (substrate)	Quantity: 62.004%<br>R-Bragg: 1.195<br>Spacegroup: Im-3m<br>Lattice parameters:<br>a = 3.1491135 Å
	$MoSe_2$	Quantity: 0.196%<br>R-Bragg: 1.619<br>Spacegroup: P63/mmc<br>Cell volume: 122.79184 Å^3

		Crystal size (Lorentzian): 9.549 nm<br>Preferred orientation (Dir: 001): 0.4411213<br>Lattice parameters:<br>a = 3.3 Å<br>c = 13.02 Å
	Se	Quantity: 0.142%<br>R-Bragg: 0.983<br>Spacegroup: Pm-3m<br>Preferred orientation (Dir: 110): 0.8828301<br>Lattice parameters:<br>a = 2.9784900 Å
0.48	Sb_2Se_3	Quantity: 40.426%<br>R-Bragg: 1.854<br>Spacegroup: Pbnm<br>Cell volume: 543.41766 $Å^3$<br>Crystal size (Lorentzian): 230.515 nm<br>Strain e0: 0.00024<br>Lattice parameters:<br>a = 11.6242041 Å<br>b = 11.7631159 Å<br>c = 3.9741856 Å
	Mo (substrate)	Quantity: 57.135%<br>R-Bragg: 1.230<br>Spacegroup: Im-3m<br>Lattice parameters:<br>a = 3.1492026 Å
	Sb_2O_3	Quantity: 2.301%

		R-Bragg: 1.246<br>Spacegroup: Fd-3m<br>Cell volume: 1384.21145 Å^3<br>Crystal size (Lorentzian): 122.923 nm<br>Strein e0: 0.00056<br>Preferred orientation (Dir: 111): 0.4039395<br>Lattice parameters:<br>a = 11.1446768 Å
	$MoSe_2$	Quantity: 0.139%<br>R-Bragg: 1.974<br>Spacegroup: P63/mmc<br>Cell volume: 123.26339 Å^3<br>Crystal size (Lorentzian): 9.549 nm<br>Preferred orientation (Dir: 001): 0.444579<br>Lattice parameters:<br>a = 3.3 Å<br>c = 13.07 Å
0.44	Sb_2Se_3	Quantity: 41.121%<br>R-Bragg: 2.652<br>Spacegroup: Pbnm<br>Cell volume: 544.06830 Å^3<br>Crystal size (Lorentzian): 157.943 nm<br>Strain e0: 0.00015<br>Lattice parameters:<br>a = 11.6257789 Å<br>b = 11.7706404 Å<br>c = 3.9758617 Å

	Mo (substrate)	Quantity: 53.041%<br>R-Bragg: 0.903<br>Spacegroup: Im-3m<br>Lattice parameters:<br>a = 3.1459973 Å
	Sb_2O_3	Quantity: 0.034%<br>R-Bragg: 1.125<br>Spacegroup: Fd-3m<br>Cell volume: 1385.37036 $Å^3$<br>Crystal size (Lorentzian): 58.242 nm<br>Strein e0: 0.00100<br>Preferred orientation (Dir: 111): 0.4025208<br>Lattice parameters:<br>a = 11.1477862 Å
	Sb	Quantity: 5.803%<br>R-Bragg: 2.471<br>Spacegroup: R-3m<br>Cell volume: 180.92240 $Å^3$<br>Crystal size (Lorentzian): 120.117 nm<br>Lattice parameters:<br>a = 4.3038579 Å<br>c = 11.2720198 Å
0.36	Sb_2Se_3	Quantity: 35.887%<br>R-Bragg: 2.269<br>Spacegroup: Pbnm<br>Cell volume: 545.18344 $Å^3$<br>Crystal size (Lorentzian): 201.739 nm

		Strain e0: 0.00070<br>Lattice parameters:<br>a = 11.6312154 Å<br>b = 11.7726258 Å<br>c = 3.9813771 Å
	Mo (substrate)	Quantity: 50.304%<br>R-Bragg: 0.321<br>Spacegroup: Im-3m<br>Lattice parameters:<br>a = 3.1479804 Å
	Sb_2O_3	Quantity: 3.247%<br>R-Bragg: 1.348<br>Spacegroup: Fd-3m<br>Cell volume: 1385.37036 $Å^3$<br>Crystal size (Lorentzian): 58.242 nm<br>Strein e0: 0.00100<br>Preferred orientation (Dir: 111): 0.4025208<br>Lattice parameters:<br>a = 11.1477862 Å
	Sb	Quantity: 10.562%<br>R-Bragg: 4.266<br>Spacegroup: R-3m<br>Cell volume: 180.86136 $Å^3$<br>Crystal size (Lorentzian): 207.035 nm<br>Lattice parameters:<br>a = 4.30332051 Å<br>c = 11.2779736 Å

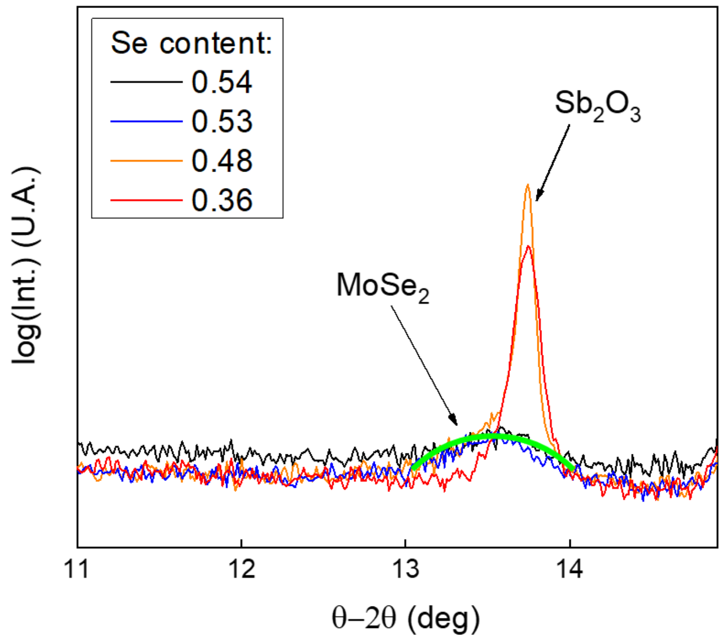

Figure S4: Enlarged diffractograms of Sb_2Se_3 thin films with 2[Se]/(2[Se] + 3[Sb]): 0.36, 0.48, 0.53, 0.54. Green mark indicates the MoSe2 (003) orientation (ICDD 00-020-0717 Powder Diffraction File patterns)

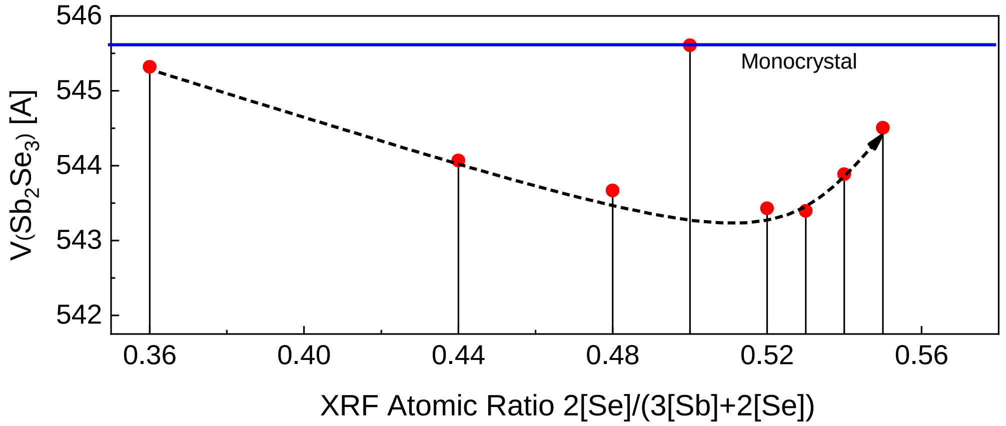

Figure S5: Evolution of cell volume as a function of 2[Se]/(2[Se] + 3[Sb])

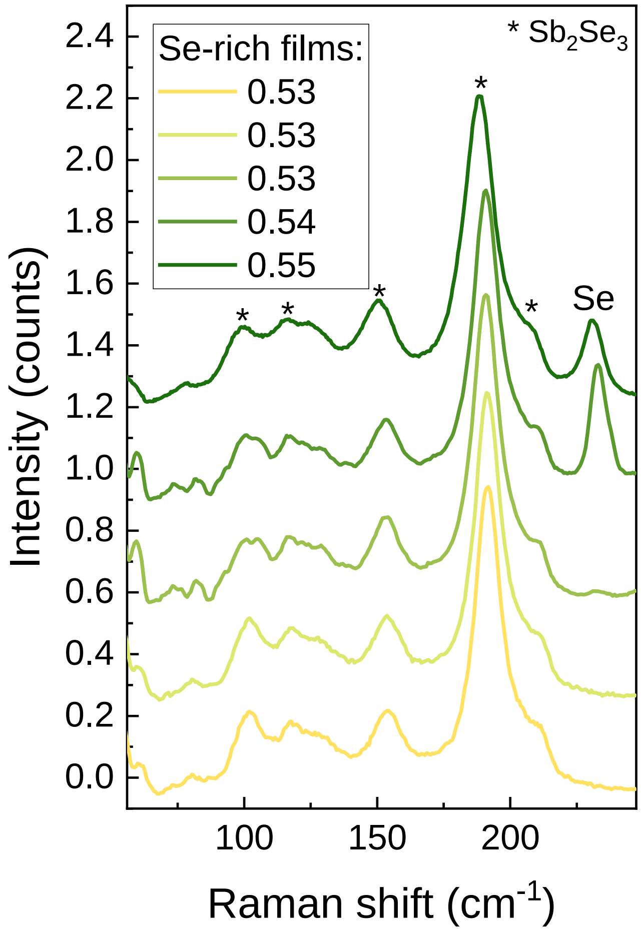

Figure S6: Complementary Raman spectra with Se phase for Se-rich samples

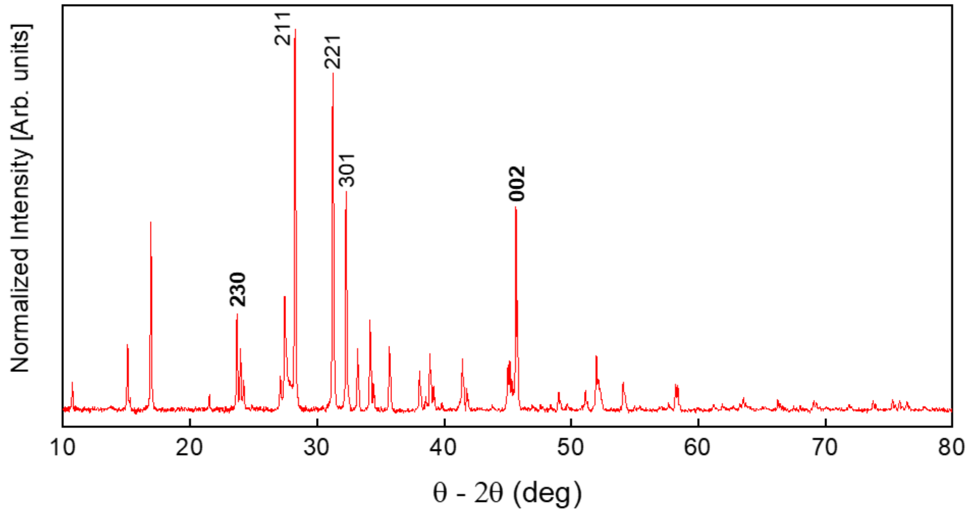

Figure S7: Complete diffractogram of a SLG/Sb_2Se_3 thin film (no Mo) prepared under the same conditions of a Se-rich SLG/Mo/Sb_2Se_3 sample

Table S2: TC values

	Bragg reflections (*hkl*)				
**Samples**	**230**	221	211	301	**002**
SLG/Sb_2Se_3	0.80	1.11	1.70	1.12	1.84
Sb-rich SLG/Mo/Sb_2Se_3	0.48	0.50	0.60	0.50	1.18
Se-rich SLG/Mo/Sb_2Se_3	0.24	0.59	1.17	1.09	4.35

Table S3: Lattice mismatch (ε) between Mo, $MoSe_2$ and Sb_2Se_3, assuming a preferred crystalline orientation in the *c* direction

**Lattice parameters (Å)**				**Mismatch (%)**
**Layers**	**a**	**b**	**c**	
Sb_2Se_3	11.62	11.72	3.962	
$MoSe_2$	2.289		12.927	8.05
Mo			3.1472	25.89

REFERENCES

(1) Boccara, A. C.; Fournier, D.; Badoz, J. Thermo-Optical Spectroscopy: Detection by the “Mirage Effect.” *Appl. Phys. Lett.* **1980**, *36* (2), 130–132. https://doi.org/10.1063/1.91395.

(2) Jackson, W. B.; Amer, N. M.; Boccara, A. C.; Fournier, D. Photothermal Deflection Spectroscopy and Detection. *Appl. Opt.* **1981**, *20* (8), 1333. https://doi.org/10.1364/ao.20.001333.

(3) Boccara, A. C.; Jackson, W.; Amer, N. M.; Fournier, D. Sensitive Photothermal Deflection Technique for Measuring Absorption in Optically Thin Media: Erratum. *Opt. Lett.* **1981**, *6* (1), 51. https://doi.org/10.1364/ol.6.000051.
